# Novel Cost-Efficient Graphene-Based Impedance Biosensor for the Analysis of Viral Cytopathogenicity and the Effect of Antiviral Drugs

**DOI:** 10.3389/fbioe.2021.718889

**Published:** 2021-07-26

**Authors:** Anke Schultz, Thorsten Knoll, Andreas Urban, Herbert Schuck, Hagen von Briesen, Anja Germann, Thomas Velten

**Affiliations:** ^1^Department of Bioprocessing and Bioanalytics, Fraunhofer Institute for Biomedical Engineering IBMT, Sulzbach, Germany; ^2^AiCuris Anti-infective Cures AG, Wuppertal, Germany

**Keywords:** impedance biosensor, graphene ink, gravure printing, viral cytopathogenicity, antiviral drugs

## Abstract

Biosensors become increasingly relevant for medical diagnostics, pharmaceutical industry, and environmental technology, for example, to test new drugs easily and reliably or to detect cell growth in changing environmental conditions. Novel materials like graphene are promising candidates to produce biosensors on an industrial scale by means of printing processes. To reach this aim, methods for the reliable and automated production of electrode structures and their coating are required. We present an impedance biosensor in the format of a microtiter plate, fabricated by highly efficient roll-to-roll printing of graphene-based microstructures on large-area polymer foils. Proof-of-principle experiments show the evidence of the suitability of the printed graphene biosensors for impedance-based monitoring of viral cytopathogenicity and its inhibition in the presence of antiviral drugs. The developed system is a promising approach toward cost-efficient impedimetric biosensors for high-throughput screening in vaccine research and antiviral drug development.

## Introduction

Electrical impedance measurement is of great interest for cell-based assays, e.g., for determining cytotoxicity, cell invasion, and migration, for monitoring of virus-induced cytopathogenicity and efficacy of neutralizing antibodies as well as of antiviral drugs (Witkowski et al., 2010; Fang et al., 2011). Particularly in the field of virological applications for the development of novel vaccine candidates and for the validation of antiviral drugs, the impedance-based procedure becomes more and more important. Most beneficial, this method does not require costly detection reagents (e.g., luciferase substrate) or artificial detection systems (e.g., reporter viruses or cell lines) (Green et al., 2008; Montefiori, 2009). It allows real-time monitoring without limitations concerning end-point results (Tian et al., 2012). However, electrodes in the current systems use precious metal films, provoking high costs (Fang et al., 2011).

Printing technologies, especially roll-to-roll gravure printing, are a promising approach toward cost-effective production of impedance-based detection systems in large quantities (Grau et al., 2016). Electrically conductive inks, e.g., based on organic substances or on conductive nanoparticles, like polysilicon, zinc oxide, or carbon nanotubes, were primarily developed for applications in the field of printed electronics. However, these materials have several drawbacks concerning the chemical stability and the mobility of charge carriers (Luechinger et al., 2008; Singh et al., 2010). Currently, graphene is considered as the optimal electrode material for many biosensors (Patel et al., 2016). Commercial graphene-based inks show increased mobility of up to 95 cm^2^V^–1^s^–1^ (Torrisi et al., 2012).

In this study, we describe the development of a novel multi-well impedance biosensor to analyze the viral-induced cytopathogenicity and to evaluate the effect of antiviral drugs. We present an electrically conductive graphene ink suitable for rotogravure printing to produce biosensors for impedance-based cell analysis. In previous work, we modified a commercial screen-printing graphene ink for the use in rotogravure printing. We investigated the electrical properties of different ink compositions, the geometrical aspects, e.g., of printing cylinders, electrode patterns and layer thickness, and the printing parameters (Knoll et al., 2016, 2017). Based on this extensive preliminary work, the current study focuses on the testing of the graphene ink concerning its biocompatibility, the roll-to-roll fabrication of the graphene-based biosensors, and their integration with bottomless well plates. As proof of suitability, the novel graphene-based sensors, together with an impedimetric measurement setup, were used to analyze the viral-induced cytopathogenicity and the effect of antiviral drugs. This encompasses the real-time monitoring of the growth of adherent cells and the formation of the cytopathic effect (CPE) after virus infection as well as CPE inhibition in the presence of antiviral drugs, exemplified with the model system of human immunodeficiency virus type 1 (HIV-1) in HIV-1 pseudovirus/TZM-bl system and herpes simplex virus 1 (HSV-1) in vero cells.

## Materials and Methods

### Cells

For biocompatibility testing, MRC-5 cells were utilized within the direct contact test and the following 10 cell lines for viability assays: J774A.1, HepG2, N18TG2, H9c2, NRK-52E, HuH-7, Vero, BALB/3T3 clone A31, NHDF, and H9. 293T/17 cells were used for preparation of the HIV-1 Env-pseudotyped virus stock (PV), vero cells for the HSV-1 virus stock, and TZM-bl cells for titration and virus inhibition assay of the PV. See details in the Supplementary Material.

### Viruses

The HIV-1 Env-pseudotyped virus stock PVO.4 (subtype B) was generated in 293T/17 cells and characterized *via* titration as well as *via* parallel-performed neutralization assays within the TZM-bl cells according to the methods described before (Schultz et al., 2012; Sarzotti-Kelsoe et al., 2014).

The HSV-1 strain SC16 had been plaque purified three times, given SC16 cl-2. The virus stock with titer of 5.21 × 10^7^ IFU/ml used in this study was generated by infecting vero cells and using the HSV-1 plaque reduction assay as described previously for titration (Biswas et al., 2014).

### Biocompatibility Testing of a Modified Graphene Ink

Three different compositions of the modified graphene ink HDPlas IGSC02002 (Haydale Ltd., United Kingdom) were tested concerning their biocompatibility. The compositions varied in the solvent used to reduce the viscosity and included the testing of acetone, DMSO, and diacetone. The qualitative direct contact test comprised of the live/dead staining with fluorescein diacetate (FDA) and propidium iodide (PI) of the indicator cell line MRC-5 and was performed in accordance with DIN ISO 10993-5 “tests for *in vitro* cytotoxicity.” Different graphene ink compositions were printed on a PET-foil and cured. Furthermore, a defined number of samples coated with Matrigel or type IV collagen were prepared to investigate the influence of protein coatings on the adherence between MRC-5 cells and the printed patterns (see details in the Supplementary Material).

To investigate the possible toxicity of the graphene on mammalian cells, PET foils printed with different densities of the graphene ink were incubated for 24 h in a culture medium. These conditioned media were then used in a cell viability assay with cells from 10 different cell lines and the AlamarBlue^TM^ reagent (details in Supplementary Material). Viability was compared with the viability of untreated cells and cells treated with 0.01 to 33-μM cycloheximide.

In order to test the influence of the graphene itself on the antiviral activity by interacting with the antiviral compounds, dilution series of four different antiviral compounds (entecavir, BAY 41-4109, efavirenz, and AZT) were preincubated for 24 h at their final assay concentrations in the presence of graphene-printed foils before they were used in standard antiviral assays.

### Standard Antiviral Assays

The influence of the graphene itself on the antiviral activity by interacting with the antiviral compounds was analyzed. Half-logarithmic dilution series from different antiviral compounds were preincubated for 24 h at their final assay concentrations in a 1-ml assay medium in the presence of 1 cm^2^ of graphene-printed foils. Then they were used to perform standard drug sensitivity assays for HIV (Wildum et al., 2013) and HBV (Corcuera et al., 2018) with MT4 and HepAD38 cells (Ladner et al., 1997), respectively.

### Fabrication of Graphene-Based Biosensors

**FIGURE 1:**
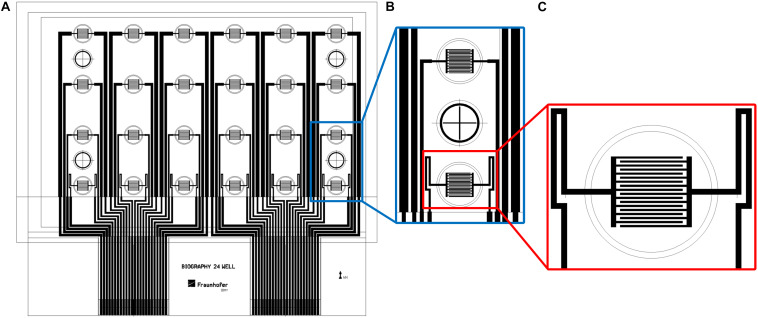
The layout of the biosensor in a well-plate format. **(A)** Drawing of complete sensor foil in a well-plate format. **(B)** Interdigitated electrode structures in two wells with one empty well in between. **(C)** Close-up view of interdigitated electrode structures. Diameter of outer circle: 7 mm.

Printing of the biosensors was conducted on a roll-to-roll gravure printing system (Saueressig GmbH, Germany). It uses 300-mm wide printing cylinders and comprises a module for corona treatment of the substrate prior to printing. Drying of the printed patterns is done by an integrated near-infrared (NIR) drying unit. For printing, we used a modification of the commercial graphene ink HDPlas IGSC02002 (Haydale Ltd., United Kingdom) and added 25 wt.% acetone for viscosity regulation. The biosensor patterns were printed on an endless 50-μm-thick PET foil (Isopet OAN, Strohmeier + Ernst GmbH, Germany) at a speed of 40 m/min. The patterns comprised interdigitated electrodes for 24 wells in a standard 96-well plate. Due to the achievable line width and resolution during roll-to-roll printing, 24 of the 96 wells were equipped with the electrode structures. The other wells were not used for the biological experiments since the conductor paths for the electrodes in the active wells were running through them. For each of the 24 active wells with a growth area of 0.3 cm^2^, an interdigitated electrode array consisted of 18 fingers with 100-μm width and 100-μm distance between the fingers. Figure 1 shows the layout of the biosensor foils with the finger structures in 24 wells of a 96-well plate.

The printed impedance sensors were cut out of the endless PET film. The higher resistivity of the gravure-printed structures compared with screen-printed electrodes was described in Knoll et al., 2017. To increase the electrical conductivity of the tracks leading to the electrodes, thin film gold tracks were added to the graphene tracks by sputtering through a metal shadow mask. The sensor foils were then assembled to bottomless 96-well plates (Greiner Bio-One GmbH, Germany) as follows: First, a medical grade double-sided adhesive film (AR 90106, Adhesives Research, Ireland) was glued to the bottomless well plate. This film was mechanically removed from those 24 wells, which were later used for cultivation and analysis. Subsequently, the PET film with the printed electrode structures was bonded to the adhesive film to form the bottom of the well plate. Finally, the printed contact pads of the impedance sensor foil were connected with a zero insertion force connector (ZIF) for the later electrical connection to the measurement system.

### Impedance Measurement System and Evaluation of Impedance Data

Analogous to commercial systems like, e.g., the xCELLigence system (Witkowski et al., 2010; Witzel et al., 2015; Lebourgeois et al., 2018), the processing of the measured impedance changes allows various evaluation strategies. In this work, the impedance curves were evaluated by means of the slope at certain time intervals and the time required to halve the impedance maximum. These examples show that the printed sensor foils are suitable for evaluation of the cytopathic effect in the presence of the applied viruses and antiviral substances.

The impedance measurement system comprised of a precision LCR meter (HP 4284A) and a switch (Keithley 7001) with a multiplexer card (Keithley 7011) that enables the parallel measurement of maximum 40 channels. The entire setup with the two sensor plates and the measurement system was connected to a computer with a LabView program for measurement control and data acquisition. All measurements were performed at a frequency of 6.3 kHz with 5 mV amplitude.

Two hours after addition of the cells, all impedance curves were set to zero to exclude any influences, like temperature changes during handling of the well plates. For ease of presentation, this time point is later referred to as “*t* = 0.” During the measurement, the liquid in each well evaporates over time due to the temperature of 37°C in the incubator. This leads to a higher concentration of ions in the conductive substance over the electrodes and thus to a permanent slight decrease in impedance. The slope of the impedance curve over time measured in wells, containing a cell medium only, was, therefore, corrected and set to zero. This correction was then also applied to all curves measured in the other wells with the test samples. Due to the different situations in each well, we only considered the impedance change for each sample and not the absolute values.

Starting with the time when the test substance was added, the impedance curves were analyzed with regard to their minima and maxima and the slope between these points. A positive slope of the impedance change represents cell proliferation and increasing density of the cell layer leading to a maximum of the impedance value after a certain time. The negative slope of the impedance curve illustrates damage and dying of the cells. Considering these characteristic slopes, we calculated a value representing the extent of the cytopathic effect. The difference of the two slopes around the maximum value is divided by the slope of the increasing curve in order to obtain a normalized value:

$$E\,x\,t\,e\,n\,t\,o\,f\,C\,P\,E=\frac{S\,l\,o\,p\,e_{\text{increase}}-S\,l\,o\,p\,e_{\text{decrease}}}{S\,l\,o\,p\,e_{\text{increase}}}$$

### Suitability Testing of New Graphene Biosensors: Impedance Measurements in Cytopathogenesis Analysis

#### Investigation of Antiviral Effect of Pritelivir on HSV-1-Infected Vero Cells

The suitability of the graphene-based biosensor was evaluated, using HSV-1 infected vero cells and pritelivir, a potent antiviral drug with an EC50 and an EC90 value of 0.01 and 0.03 μM, respectively, against HSV-1 SC16 cl-2 (Biswas et al., 2014). The impedance signal was recorded for 5 days post infection. See further details in the Supplementary Material.

#### Investigation of Antiviral Effect of Efavirenz on HIV-1-Infected TZM-bl Cells

TZM-bl-infected HIV-1 Env-pseudotyped cells and efavirenz were used to further evaluate the graphene-based biosensors. Efavirenz is a non-nucleoside reverse transcriptase inhibitor of HIV-1 (Young et al., 1995) and was obtained through the NIH AIDS Reagent Program, Division of AIDS, NIAID, NIH. The antiviral effect of efavirenz was finally tested by preparing the cell samples as described in the Supplementary Material. Infection was performed with the HIV-1 Env-pseudotyped virus PVO.4 plus 5-μg DEAE-Dextran according to the procedure described in Sarzotti-Kelsoe et al., 2014, with a final dilution of 1:10 or within a five-fold limiting dilution row from 1:10 to 1:1,250 in the presence or absence of efavirenz (1 or 2 μM). Impedance was measured during 72 h after addition of the cells. Measurements were performed every 7 min at 6.3 kHz/5 mV and evaluated as described above. Cell confluence was documented, using the microscope DMI4000. See further details in the Supplementary Material.

## Results and Discussion

### Biocompatibility Studies of the Graphene Ink

The results of the direct contact test showed no incidences for cell cytotoxicity 48 h after seeding of the MRC 5 cells on the foil substrate samples (see Supplementary Figure 1). They showed good viability (green fluorescence) with nearly no dead cells (red fluorescence). The biocompatibility of the graphene ink was validated successfully for the pure graphene and for the graphene dissolved in acetone, DMSO, or diacetone. However, the adherence of the MRC-5 fibroblasts on the printed graphene structures was reduced. Additional coating of the graphene-printed PET foil substrates with proteins, especially Matrigel (80 μg/ml) improved cell adhesion. Thus, this treatment was applied for the following experiments. Acetone was finally selected for the roll-to-roll fabrication of the graphene biosensors.

**FIGURE 2:**
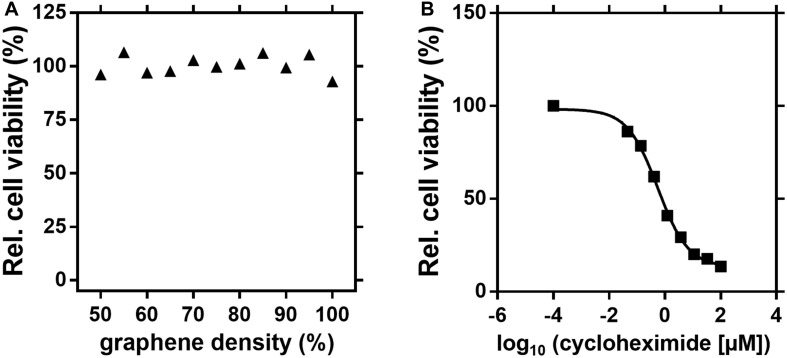
No influence of the graphene ink on the cell viability of HuH-7 cells. About 1-cm^2^ pieces of graphene ink-printed PET-foil in different densities, ranging from 50 to 100%, were preincubated for 24 h in a culture medium. Subsequently, a cell viability assay with the AlamarBlue^TM^ reagent was performed with these conditioned media. An effect of graphene-conditioned media on cell viability of HuH-7 cells was not observed **(A)**. In a control experiment with fresh media and different concentrations of cycloheximide, the cytotoxic agent showed a dose-dependent effect on the viability of HuH-7 cells with a calculated CC50 value of approximately 0.6 μM **(B)**.

As shown for HuH-7 cells in Figure 2, using the concept of graphene-conditioned media, no influence of graphene on the viability of cells was observed. In contrast, it was possible to demonstrate the dose-dependent cytotoxicity of cycloheximide with a calculated CC50 value of 0.6 μM (Figure 2B). No cytotoxic effects of graphene could be demonstrated for the other nine mammalian cell lines (data not shown).

**FIGURE 3:**
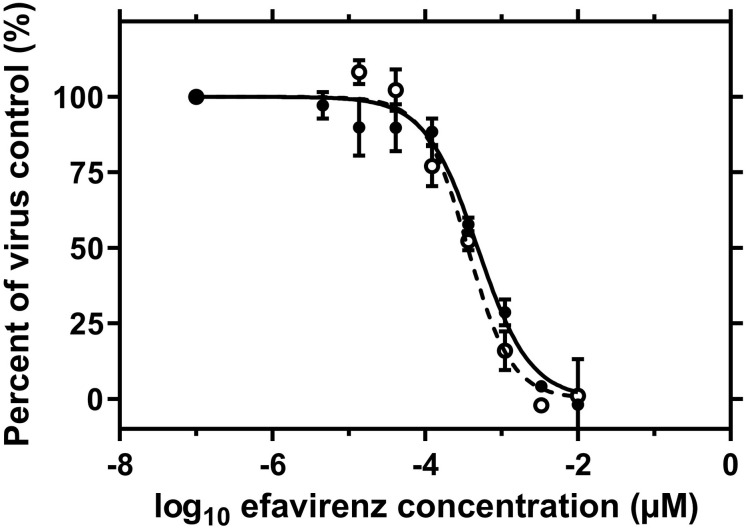
No influence of the graphene ink on the dose-dependent antiviral activity of efavirenz in a cell-based HIV replication assay. An 8-point half-logarithmic serial dilution of efavirenz, starting from 0.01 μM, was preincubated for 24 h with (a solid line) or without (a dashed line) graphene ink on PET-foil before use in a standard antiviral assay. The preincubation of efavirenz with graphene did not change the EC50 value of approximately 0.4 nM nor the overall characteristics of the dose-response curve. Error bars represent the standard deviation of triplicate samples.

The use of an efavirenz dilution series, preincubated with graphene in a standard antiviral assay, showed that graphene did not change the antiviral activity (Figure 3). This result was verified for AZT as another inhibitor of HIV replication and for BAY 41-4109 and entecavir as two different inhibitors of HBV (data not shown).

### Fabrication and Set-Up of Printed Impedance Biosensors for the Application of CPE Analysis and a Virus Inhibition Assay

**FIGURE 4:**
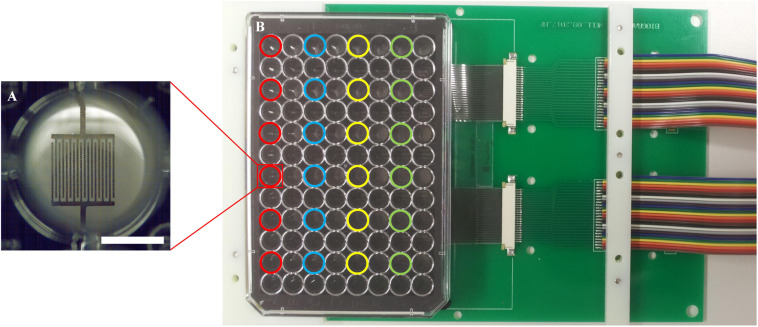
Measurement set-up. **(A)** Close-up view of one of the 24 wells with sensor structures; a scale bar corresponds to 3.5 mm. **(B)** A well plate with sensor foil and interdigitated graphene sensors, connected with a circuit board. Different colors highlight the active wells with electrodes; each color represents one row with six wells, which are used in parallel.

Two 96-well plates with impedance sensors at the bottom of each of the 24 active wells in were prepared for parallel measurement of samples in a standard lab incubator. Twelve samples were measured in parallel in each experiment, i.e., six wells per plate were operated during one measurement. These six active wells per plate were located in the same row to exclude any influence of different lengths of the conductor paths on the measurement (Figure 4). The plates were connected to the impedance measuring system, cells were seeded into the wells, and the circuit boards with the sensor well plates were placed inside the incubator. Automated impedance measurement during cultivation was performed *via* the electrical interface between the circuit board in the incubator and the impedance measuring system outside.

### Suitability Testing of New Graphene Biosensors: Impedance Measurements in Cytopathogenesis Analysis

#### Investigation of Antiviral Effect of Pritelivir on HSV-1-Infected Vero Cells

**FIGURE 5:**
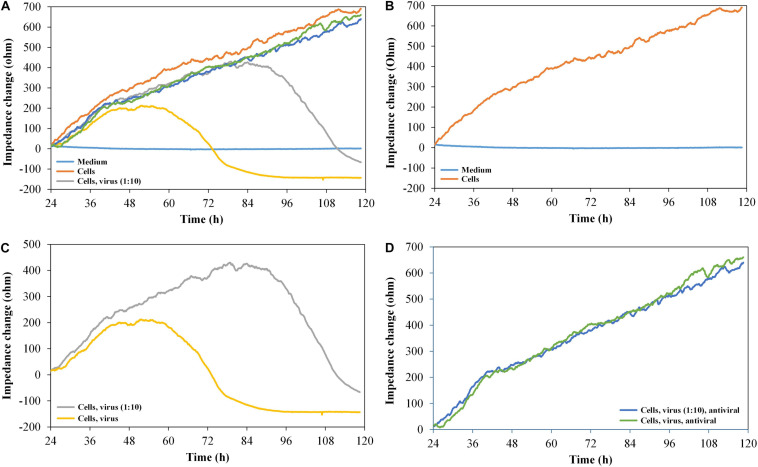
Impedance change curves of vero cells, exposed to HSV-1 and antiviral substance pritelivir, *n* = 2. **(A)** Impedance curves of all six channels. **(B–D)** show two selected curves each from **(A)**. **(B)** Medium control and cells without virus. **(C)** Cells with 500 IFU/ml of HSV-1 SC16 cl-2 virus and 1:10 diluted virus. **(D)** Cells with virus, undiluted (500 IFU/ml) and 1:10 diluted in the presence of 1 μM of the antiviral agent pritelivir. All the curves were set to 0 Ω at *t* = 24 h (infection with HSV-1/addition of pritelivir). The slope of medium average was corrected to 0, and all other curves were modified correspondingly.

To verify the suitability of the printed graphene-based biosensor to analyze the cytopathogenicity caused by viral replication, vero cells were seeded into the sensor plate and were subsequently infected with HSV-1 after 24 h for cell attachment (Figure 5).

As expected, the impedance signal for non-infected vero cells increased over time. The cells infected with 500 IFU/ml of HSV-1 SC16 cl-2 first generated an increasing impedance signal and then reached a plateau approximately 24 h after infection. The signal dropped for another 24 h until it reached the base line level again. The reduction in the impedance signal clearly reflected the CPE typical for HSV-1 on vero cells. The cells infected with only 50 IFU/ml showed similar impedance kinetics, but the plateau of high signal was reached later (approximately at 2.5 days post infection). However, the signal drop after the plateau was of the same magnitude as observed for the cells infected with the high titre. After demonstrating that the infection kinetics of HSV-1 on vero cells can be monitored with the graphene-based impedance biosensor, the effect of an antiviral agent on virus-infected cells was analyzed. For this test, we used pritelivir, a highly potent inhibitor of HSV replication (Kleymann et al., 2002). Figure 5D shows that the presence of pritelivir at a concentration of 1 μM, protected the cells from the CPE and cell lysis. A decrease in the impedance signal after a certain time of virus replication as seen for the untreated control cells (Figure 5C) could not be determined for pritelivir treated cells. This clearly demonstrates that the graphene-based biosensor could be a strong tool in the attempt to screen for novel antiviral agents.

#### Investigation of Antiviral Effect of Efavirenz on HIV-1-Infected TZM-bl Cells

**FIGURE 6:**
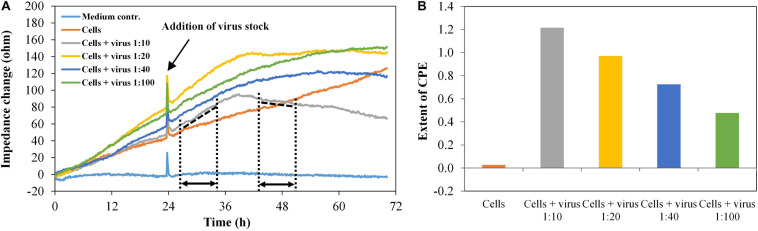
TZM-bl cells, exposed to different virus concentrations, *n* = 2. **(A)** Impedance curves. All curves were set to 0 Ω at *t* = 0. The slope of the medium average was corrected to zero, and all other curves were modified correspondingly. The dashed and dotted lines in the diagram illustrate the curve regions used to calculate the extent of the cytopathic effect (CPE). **(B)** Extent of CPE as a function of virus concentration. Values were calculated, using the time intervals illustrated in **(A)** and the formula described in section “Fabrication of Graphene-Based Biosensors.”

Infection experiments were performed in duplicate with the HIV-1 Env-pseudotyped virus PVO.4, which was pre-diluted corresponding to “high-titer” (1:10), “middle-titer” (1:20 and 1:40), and “low-titer” virus (1:100). Figure 6A shows the impedance curves of the samples.

The impedance measurement started with the addition of cells (*t* = 0). In the wells containing the cells without virus, the impedance values increase continuously as the cells grow to a confluent cell monolayer. In the wells with the infected cells, the impedance signal increases to a maximum within 16 h post infection (time interval, 26.5–35 h), which is typical for the cell growth phase. In the time interval 35–43.5 h, the slope of the impedance curve changes from positive to negative values. Finally, the curve decreases constantly, enabling the real-time monitoring of the virus-induced CPE formation in different intensities, which depend on the virus dilution of PVO.4 (time interval 43.5–51.5 h). For each channel, the extent of the cytopathic effect (CPE) was calculated, using the formula given above.

The values in Figure 6B facilitate the direct comparison of the assay set-up in two separate sensor plates. The only slight differences in values measured in the two different well plates indicate that the graphene-based biosensor plates enable reproducible measurements. Furthermore, it is possible to distinguish between different virus infectivities. About 48 h after infection, the assay was stopped to perform a microscopic analysis of the cells. The cells at the highest virus concentration (1:10 final dilution in the well) showed only 10% cell confluence. In the sample with 1:100 final virus dilution, 80% of the sensor plate were covered with TZM-bl cells (see Supplementary Figure 2). These optical results confirmed the results from the impedance measurements.

**FIGURE 7:**
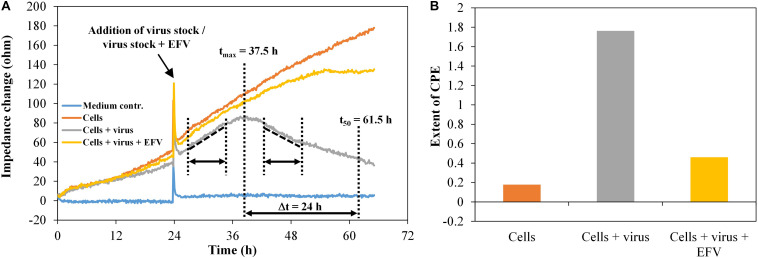
TZM-bl cells, exposed to HIV-1 pseudovirus and antiviral substance efavirenz (1 μM), *n* = 2. **(A)** Impedance curves. All curves were set to 0 Ω at *t* = 0. The slope of medium average was corrected to 0, and all other curves were modified correspondingly. The dashed and dotted lines in the diagram illustrate the curve regions used to calculate the extent of the cytopathic effect. **(B)** Extent of CPE without and with antiviral substance efavirenz. Values were calculated, using the time intervals illustrated in **(A)** and the formula described in section “Fabrication of Graphene-Based Biosensors.”

To evaluate the virus inhibition assay, impedance data were collected for the model systems HIV-1 in the presence of the antiretroviral substance efavirenz (EFV). The HIV-1 pseudovirus stock PVO.4 was used with a constant final concentration per well of 1:10 plus 1-μM EFV, as well as the HIV-1 pseudovirus stock five-fold serial diluted from 1:10 to 1:1,250, utilizing EFV at a concentration of 2 μM. As illustrated in Figure 7A, the change in the slope of the impedance curve was again used as a measure for the extent of the cytopathic effect.

TZM-bl cells showed a typical cell growth indicated by a continuous increase of the impedance values (Figure 7A). The addition of the HIV-1 pseudovirus stock plus EFV hardly changes this behavior, since the antiviral substance largely inhibits virus replication and, in consequence, the formation of cytopathogenicity. The infected cells showed an impedance signal increase with a constant rise in the time interval 26.5–35 h, followed by a decrease, representing the viral effect on the cell monolayer in the time interval 43.5–51.5 h. We also determined the time after which the number of cells exposed to the virus reached 50% of the maximum. This was the case after around 24 h. During the same time, the cell number increased to 157% in wells only with cells and to 135% in wells with cells plus virus plus EFV. For each well, the extent of the cytopathogenicity [cytopathic effect (CPE)] was calculated. The detection of the cytopathogenicity strength and its inhibition by 1-μM EFV was reproducible and was ±0.05 in the wells containing the pseudovirus PVO.4 and ±0.13 for the samples containing 1-μM EFV (Figure 7B).

The microscopic assessment confirmed the data determined *via* impedance measurement with a strong cytopathic effect in infected samples (10% confluence) and an inhibition of the CPE in the presence of 1-μM EFV (80% confluence) (Supplementary Figure 3).

**FIGURE 8:**
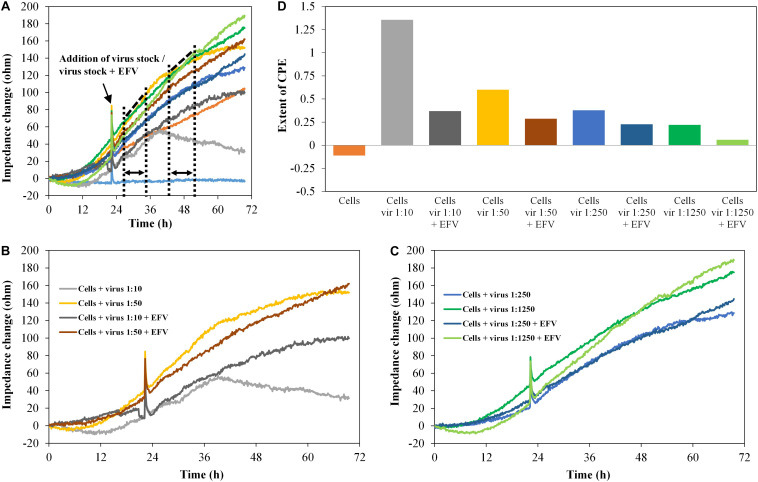
TZM-bl cells, exposed to HIV-1 pseudovirus in different concentrations and antiviral substance efavirenz (2 μM), *n* = 2. **(A–C)** Impedance curves. All curves were set to 0 Ω at *t* = 0. The slope of medium average was corrected to 0, and all other curves were modified correspondingly. **(B,C)** show four curves each from **(A)**. The dashed and dotted lines in **(A)** illustrate the curve regions used to calculate the extent of the cytopathic effect. **(B)** shows the samples with high virus concentrations 1:10 and 1:50, without and with EFV, **(C)** shows the samples with low virus concentrations, 1:250 and 1:1,250, without and with EFV. **(D)** Extent of CPE on cells with different virus concentrations and without/with antiviral substance. Values were calculated, using the formula described in section “Fabrication of Graphene-Based Biosensors.”

The diagrams in Figure 8 show the impedance characteristics after addition of the pseudovirus stock PVO.4 in four different concentrations (five-fold dilution series from 1:10 to 1:1,250). Figures 8B,C show four curves, each from the upper diagram (Figure 8A).

Increasing virus concentration enhances the virus-induced cytopathogenicity, which was clearly reflected by the impedance curve. In the presence of EFV (2 μM), the CPE was inhibited. This was also displayed in the impedance signal, which was reduced to a level almost identical to the cell control. The microscopic analysis again confirmed the development of the CPE and its inhibition by EFV (data not shown).

## Conclusion

There is a strong need for low-cost high-throughput-capable readout systems to evaluate neutralizing antibody responses of promising vaccine candidates as well as to identify and validate new antiviral substances. This need is also emphasized by the current COVID-19 pandemic. Our presented process for the cost-effective production of graphene-based biosensors by roll-to-roll printing on an industrial scale is the optimal basis for providing low-cost impedimetric sensors for such high-throughput screenings. Furthermore, the demonstration of the applicability for virological applications in the context of the virus inhibition assay constitutes the importance of our biosensors for the development of vaccines and antiviral drugs.

Altogether, our novel graphene-based biosensors hold great promise to facilitate and support the vaccine research and antiviral drug discovery in upcoming preclinical and clinical trials.

After the successful proof-of-principle experiments presented in this work, we will carry out further test series regarding selectivity and reproducibility and consider biological factors like cultivation time and different cell types as well as technological factors in optimization of the sensor characteristics and system performance. Although they are not directly comparable, we would like to contrast the results obtained with our system with commercial systems. The impedance spectroscopy system from the German company nanoAnalytics, for example, uses reusable measurement plates, comprising 24 wells (Benson et al., 2013). In contrast to our sensor plate, the position of the electrodes in the commercial system does not allow for the optical control of the cells during the measurement. Furthermore, in contrast to the above-mentioned commercial system, our single-use device does not require long preparation and cleaning procedures.

The sensor plates presented in this work use 24 wells of a 96-well plate. There are commercial systems available, which can use all 96 wells of a plate for recording. An example is the commercial electrical cell impedance spectroscopy (ECIS) system from Applied Biophysics (Pennington and Van de Walle, 2017). This commercial system, however, is a real product and thus not comparable with the system we used for our proof-of-principle experiments. We have shown that printed electrodes made of graphene are compatible with the processes and substances used in neutralizing antibody response evaluation. With respect to the final goal of a real product, a redesign of the electrodes and conductive traces, taking into account improved ink configurations (e.g., with varied graphene concentration), can lead to further optimization of the manufacturing process. In the long run, this could lead to a low-cost device, thanks to cost-efficient fabrication *via* roll-to-roll printing. Further optimization is, therefore, necessary with regard to the future mass production of the sensor foils. For example, multilayer printing or combination of inks with different conductivities, either as, e.g., a graphene-silver composite (Karim et al., 2019), or using different inks for electrodes and conductor paths can increase the electrical conductivity and avoid the additional time-consuming and expensive sputtering process.

## Data Availability Statement

The raw data supporting the conclusions of this article will be made available by the authors, without undue reservation.

## Author Contributions

AS, AU, HB, and AG contributed to conception, design, and validation of the study. HB and TV supervised the study. AS and AU performed the investigation. HS performed the formal analysis. HS, AU, and TK coordinated the visualization of results. AS and TK wrote the first draft of the manuscript. TV and AU wrote the sections of the manuscript. All the authors contributed to the manuscript revision, and read and approved the submitted version.

## Conflict of Interest

AU is an employee of AiCuris Anti-infective Cures AG, the company that is developing pritelivir. The remaining authors declare that the research was conducted in the absence of any commercial or financial relationships that could be construed as a potential conflict of interest.

## Publisher’s Note

All claims expressed in this article are solely those of the authors and do not necessarily represent those of their affiliated organizations, or those of the publisher, the editors and the reviewers. Any product that may be evaluated in this article, or claim that may be made by its manufacturer, is not guaranteed or endorsed by the publisher.
