# Supplementary material for: Novel Cost-Efficient Graphene-Based Impedance Biosensor for the Analysis of Viral Cytopathogenicity and the Effect of Antiviral Drugs
Source: Front Bioeng Biotechnol. 2021 Jul 26;9:718889. doi: 10.3389/fbioe.2021.718889 (PMC8350578; doi:10.3389/fbioe.2021.718889)
Supplement: Supplementary file 1 [file Data_Sheet_1.PDF]

# *Supplementary Material*

## 1 Material and Methods

### 1.1 Cells

**Supplementary Table 1. Cell lines used (in alphabetical order)**

| ***Cell line*** | ***Ordering information*** |
|---|---|
| BALB/3T3 | ATCC® CCL-163™ |
| HepAD38 | Fox Chase Cancer Center, Rockwell, USA (Corcuera, A. et al., 2018) |
| HepG2 | ATCC® HB-8065™ |
| H9 | ATCC® HTB-176™ |
| H9c2 | ATCC® CRL-1446™ |
| HuH-7 | JCRB Cell Bank® JCRB0403™ |
| J774A.1 | ATCC® TIB-67™ |
| MRC-5 | ATCC® CCL171™ |
| MT4 | NIH AIDS Research and Reference Reagent Program |
| NHDF | Lonza #CC-2511 |
| NRK-52E | ATCC® CRL-1571™ |
| N18TG2 | DSMZ ACC 103™ |
| TZM-bl | NIH AIDS Research and Reference Reagent Program, catalog no. 8129 |
| Vero | ATCC® CCL-81™) |
| 293T/17 | ATCC® CRL-11268™ |

The MRC-5 cells were cultured in MEM α, Glutamax medium (Gibco™, Thermo Fisher Scientific Waltham, MA, USA) containing 10 % FBS (Sigma-Aldrich, Merck, Darmstadt, Germany) as well as penicillin 100 µg/ml and streptomycin 100 U (Gibco™, Thermo Fisher Scientific Waltham, MA, USA) and were split 1:3 twice weekly. The 293T/17 and TZM-bl cells were cultivated as previously described (Schultz et al., 2012, Sarzotti-Kelsoe, M. et al., 2014).

J774A.1, HepG2, N18TG2, H9c2, NRK-52E, HuH-7 and Vero cells were cultured in DMEM (PAN Biotech, Aidenbach, Germany) containing 1 mM Na-pyruvat, 2 mM L-glutamine, penicillin 100 µg/ml and streptomycin 100 U (all from Gibco™, Thermo Fisher Scientific Waltham, MA, USA) and 10 % FBS (Biochrom, Berlin, Germany). BALB/3T3 clone A31 and NHDF cells were cultured in MEM containing 1 mM Na-pyruvat, 2 mM L-glutamine, penicillin 100 µg/ml and streptomycin 100 U, 10 mM HEPES (all from Gibco™, Thermo Fisher Scientific Waltham, MA, USA) and 10 % FBS (Biochrom, Berlin, Germany). H9 cells were cultured in RPMI (PAN Biotech, Aidenbach, Germany) containing 2 mM L-glutamine, penicillin 100 µg/ml and streptomycin 100 U (Gibco™, Thermo Fisher Scientific Waltham, MA, USA) and 10 % FBS (Biochrom, Berlin, Germany).

## 1.2 Biocompatibility testing of modified graphene ink

### 1.2.1 Direct contact tests

A PET-foil sample with an area of 1 cm² was fixed with Elastosil® E43 (Wacker Silicones, Drawin-Vertriebs GmbH Ottobrunn, Germany) known for its biocompatibility in central position at the bottom of a 35 mm petri dish (Greiner bio-one, Kremsmünster, Austria) and sterilized with ethylene oxide. For protein coating the samples were incubated with 0.1 mg/ml DQ™ collagen type IV from human placenta (Invitrogen™, Thermo Fisher Scientific Waltham, MA, USA) or 80 µg/ml Corning® Matrigel® (Corning Life Science, Corning, New York, USA) for 4 h at 37 °C, 5 % $CO_2$ and 95 % humidity. 1 x $10^5$ MRC-5 cells were applied per 35 mm petri dish and stained 48 h post seeding with 30 µg/ml FDA (Sigma-Aldrich, Merck, Darmstadt, Germany) and 40 µg/ml PI (Sigma-Aldrich, Merck, Darmstadt, Germany) for 15 s. After rinsing with Dulbecco's Phosphate Buffered Saline (DPBS) (Gibco™, Thermo Fisher Scientific Waltham, MA, USA), samples were immediately analyzed with an inverted fluorescence microscope (Olympus IX70, Olympus Germany GmbH, Hamburg, Germany) using Paint Shop Pro 5 for image processing. A total of nine different sample groups were analyzed within this biocompatibility study: (a) PET-foil substrate as biocompatible reference material, (b) pure graphene ink, (c) graphene ink plus acetone, (d) graphene ink plus DMSO, (e) graphene plus diacetone, (f) graphene ink plus type IV collagen coating, (g) graphene ink plus Matrigel coating, (h) graphene plus diacetone plus type IV collagen coating and (i) graphene plus diacetone plus Matrigel coating.

### 1.2.2 Cell viability assays

1 $cm^2$ large pieces of PET foil printed with different densities of the graphene ink were incubated for 24 h in 1 ml of the appropriate cell culture medium mentioned in section 2.1 above. Using these conditioned media, cells of ten different cell lines were seeded into 96 well plates at the following densities: 5 x $10^4$/ml for J774A, HepG2, BALB/3T3 clone A31, H9c2 and NHDF, 2.5 x $10^4$/ml for N18TG2 and NRK-52E, 3 x $10^4$/ml for HuH-7 and Vero, and 3.1 x $10^5$/ml for H9. All samples were incubated for 72 hours at 37 °C and 5 % $CO_2$. Subsequently, AlamarBlue™ reagent (BioSource International, Camarillo, USA) was added in a final concentration of 10 % to the cells, and the fluorescence signal was measured after 2 to 4 hours further incubation at 550/595 nm using a SpectraFluor Plus fluorescence reader (Tecan, Crailsheim, Germany).

## 1.3 Standard antiviral assay

### 1.3.1 Antiviral activity testing with MT4 cells

The antiviral activities of test compounds against HIV were evaluated in MT-4 cells by the alamarBlue viability assay as previously described (Wildum, S. et al., 2013). Briefly, MT-4 cells (4 x $10^5$/ml in RPMI 1640 supplemented with 2 % FCS) infected with HIV-1 LAI at a multiplicity of infection (MOI) of approximately 0.01 were added to a final volume of 100 µl per well using the conditioned medium mentioned above. After a 5-day incubation period at 37 °C and 5 % $CO_2$, 10 µl of AlamarBlue® reagent (BioSource International, Camarillo, USA) was added to each well, and the fluorescence signal was measured after 3 h at 550/595 nm using a SpectraFluor Plus fluorescence reader (Tecan, Crailsheim, Germany).

### 1.3.2 Anti-HBV activity testing with Hep38AD cells

The antiviral activities of test compounds against HBV were evaluated in HepAD38 cells (Ladner, S.K. et al., 1997) by the quantification of encapsidated HBV DNA in the cell supernatant using qPCR as previously described (Corcuera, A. et al., 2018). Briefly, 60,000 cells were seeded in Dulbecco's modified Eagle's medium / Nutrient Mixture F-12 (Gibco, Karlsruhe), 10 % fetal bovine serum (PAN Biotech, Aidenbach) into each well of a 96-well plate and treated with serial half-log dilutions of test compound pre-incubated for 24 h with or without graphene-printed foils. At day 6, HBV DNA from 100 µl 0.45 µM filtrated cell culture supernatant was purified using the MagNA Pure 96 DNA and Viral NA Small Volume Kit (Roche Diagnostics, Mannheim). To determine relative copy numbers of HBV DNA, 5 µl of the 100 µl eluate containing HBV DNA were subjected to PCR LC480 Probes Master Kit (Roche) together with 1 µM antisense primer tgcagaggtgaagcgaagtgcaca, 0.5 µM sense primer gacgtcctttgtttacgtcccgtc, 0.3 µM hybprobes acggggcgcacctctctttacgcgg-FL and LC640-ctccccgtctgtgccttctcatctgc-PH (TIBMolBiol, Berlin) to a final volume of 12.5 µl. The PCR was performed on the Light Cycler 480 real time system (Roche Diagnostics, Mannheim) using the following protocol: Pre-incubation for 1 min at 95 °C, amplification: 40 cycles with each 10 s at 95 °C, 50 s at 60 °C, 1 s at 70 °C, cooling for 10 s at 40 °C. Viral load was quantitated against known standards using plasmid DNA with an HBV insert.

## 1.4 Suitability testing of new graphene biosensors: impedance measurements in cytopathogenesis analysis

### 1.4.1 Investigation of antiviral effect of pritelivir on HSV-1 infected Vero cells

250 µl of culture medium (DMEM (PAN Biotech, Aidenbach, Germany) containing 1 mM L-glutamine, penicillin 100 µg/ml and streptomycin 100 U (Gibco™, Thermo Fisher Scientific Waltham, MA, USA) and 10 % FBS (Biochrom, Berlin, Germany) were added to each well of the sensor plate until a stable impedance signal was obtained. After that, the culture medium was replaced by a medium with a low FBS concentration of 1 % and subsequently 2.5 x $10^4$ Vero cells in a volume of 250 µl were added to the wells. After 24 h of cell attachment, 500, 50 or 0 infectious units per well of HCV-1 SC16 cl-2 were added. After 1 h of adsorption the virus was removed by washing with PBS and by adding fresh medium with or without 1 µM pritelivir.

### 1.4.2 Investigation of antiviral effect of efavirenz on HIV-1 infected TZM-bl cells

The biosensors were first incubated with 200 µl medium per well until a stable impedance signal was obtained. 1.0 x $10^4$ TZM-bl cells per Matrigel coated (80 µg/ml) well were seeded and infected after 24 h with the HIV-1 Env pseudotyped virus PVO.4 in the presence of 5 µg DEAE-Dextran (Sigma-Aldrich, Merck, Darmstadt, Germany). PVO.4 was characterized with a dilution of 1:200 at an RLU (relative luminescence units) of 150,000 representing a virus stock with high infectivity. To simulate the existence of middle and low titer viruses, PVO.4 was pre-diluted in medium 1:2, 1:4 and 1:10 and assayed in the final dilutions per well of 1:10 (high titer virus), 1:20 and 1:40 (middle titer virus) and 1:100 (low titer virus). Wells filled with medium served as blank control and wells with not infected cells as negative control. After reloading the plate into the incubator (37 °C, 5 % $CO_2$ and 95 % humidity), impedance was measured every 7 minutes at 6.3 kHz/5 mV for up to 48 h. Additionally, the confluence of the respective wells was documented 48 h post infection using the microscope DMI4000 (Leica, Wetzlar, Germany).

## 1.5 Software

Two formally validated Excel-based macros were utilized to determine the dilution to achieve 150,000 RLU within the titration assay and to calculate the IC50 values based on the measured luminescence data within the neutralization assay (Sarzotti-Kelsoe, M. et al., 2014). Paint Shop Pro 5 was used for image processing of the results for biocompatibility testing and Microsoft Excel 2013 for the evaluation of the impedance measurement data. Prism 6 (GraphPad Software Inc., La Jolla, USA) was used to fit a nonlinear regression curve to the data from standard antiviral and cell viability assays and to calculate half-maximal effective concentrations ($EC_{50}$).

## 2 Results and discussion

### 2.1 Biocompatibility studies of the graphene ink

#### 2.1.1 Direct contact test with MRC-5

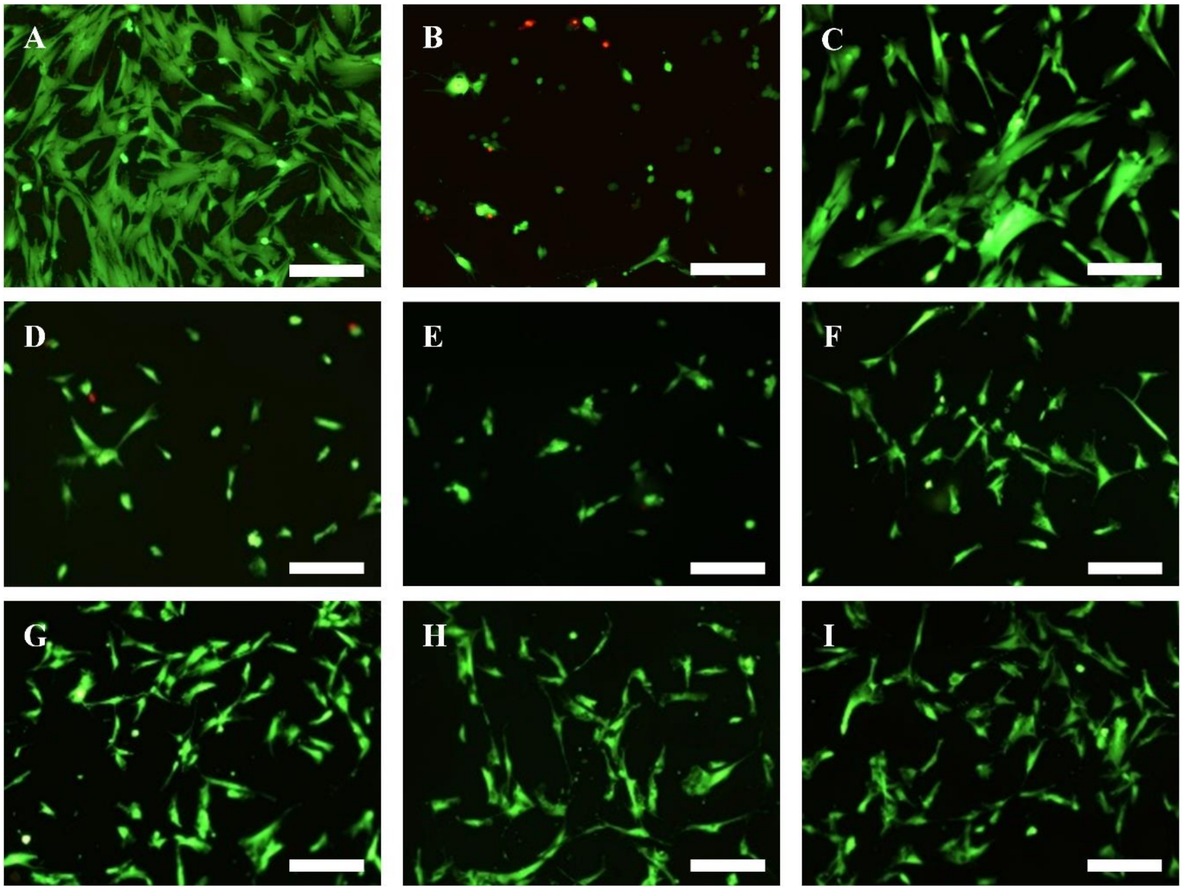


**Supplementary Figure 1. Live-dead staining of MRC-5 cells after cultivation for 48 h on PET foil substrates without and with graphene ink.** A: polystyrene plates suited for cell culture, B: polystyrene plates suited for cell culture plus 0.2 % HEMA, C: PET foil, D: pure graphene ink, E: graphene ink plus acetone, F: pure graphene ink plus type IV collagen, G: pure graphene ink plus Matrigel, H: graphene plus diacetone plus type IV collagen, I: graphene plus diacetone plus Matrigel. Green cells correspond to FDA-stained viable cells and red cells to PI-stained not viable ones. Measurement bar corresponds to 200 µm.

## 2.2 Suitability testing of new graphene biosensors: impedance measurements in cytopathogenesis analysis

### 2.2.1 Infection of TZM-bl cells with HIV-1 pseudovirus PVO.1 in different dilutions

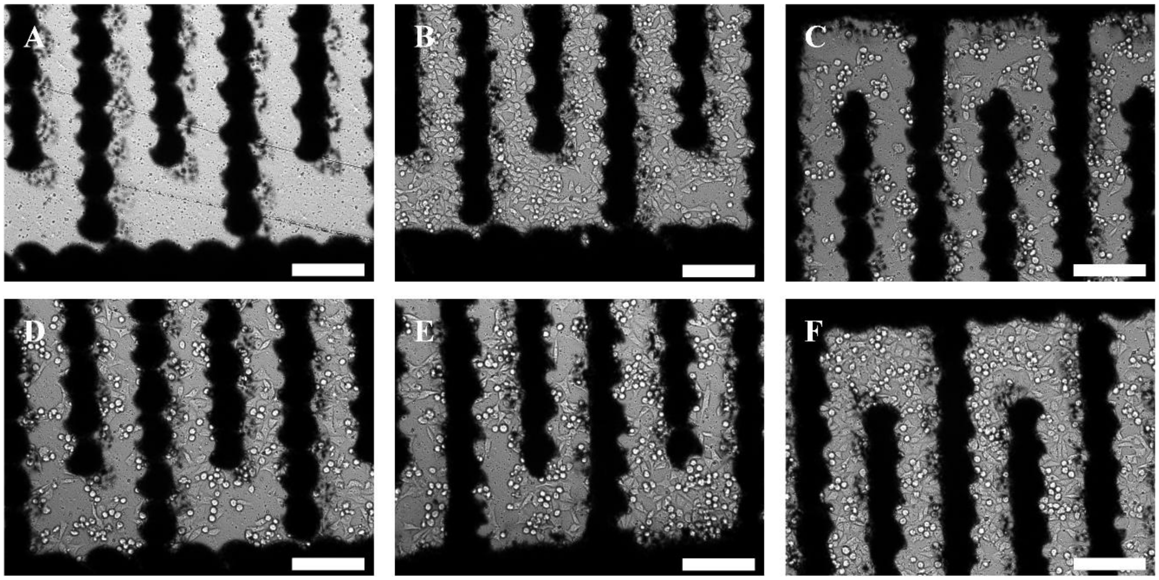


**Supplementary Figure 2. Evaluation of the confluence 48 h after infection of TZM-bl cells with HIV-1 pseudovirus PVO.1 in different dilutions.** A: Medium control, B: TZM-bl cells without virus (90 % confluent), C: TZM-bl cells plus PVO.4 finally diluted 1:10 (10 % confluent), D: TZM-bl cells plus PVO.4 finally diluted 1:20 (40 % confluent), E: TZM-bl cells plus PVO.4 finally diluted 1:40 (60 % confluent), F: TZM-bl cells plus PVO.4 finally diluted 1:100 (80 % confluent). Measurement bar corresponds to 200 µm.

### 2.2.2 Infection of TZM-bl cells with HIV-1 pseudovirus PVO.1 and addition of antiviral substance efavirenz

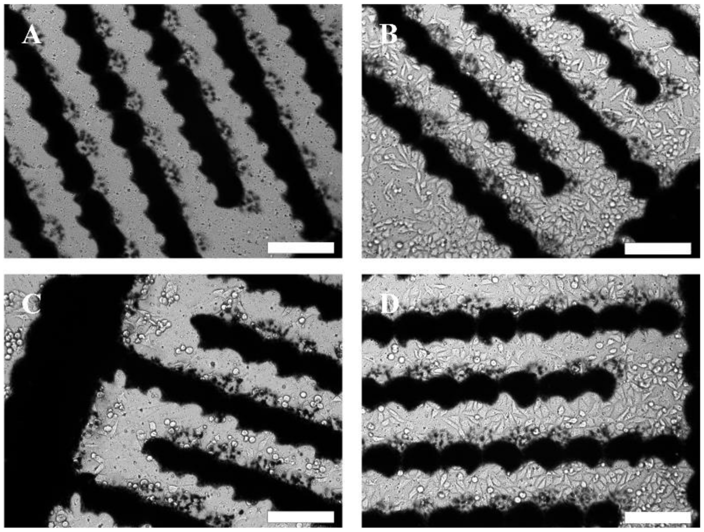


**Supplementary Figure 3. Microscopic evaluation of the wells 48 hours after infection of the TZM-bl cells with the HIV-1 pseudovirus PVO.4.** Only the areas between the graphene electrodes (black structures) can be observed microscopically. A: Wells with medium show no cells. B: TZM-bl cells of the cell control have reached 80 – 90 % confluence. C: 40 – 50 % confluence after virus infection of TZM-bl cells. D: In the presence of the antiviral substance efavirenz, 48 h after infection with the virus stock PVO.4, the cells show a confluence of about 80 %. Measurement bar corresponds to 200 µm.
